# Nanocarriers, Progenitor Cells, Combinational Approaches, and New Insights on the Retinal Therapy

**DOI:** 10.3390/ijms22041776

**Published:** 2021-02-10

**Authors:** Elham Pishavar, Hongrong Luo, Johanna Bolander, Antony Atala, Seeram Ramakrishna

**Affiliations:** 1 Pharmaceutical Research Center, Pharmaceutical Technology Institute, Mashhad University of Medical Sciences, Mashhad 91735, Iran; pishavare931@mums.ac.ir; 2 Wake Forest Institute for Regenerative Medicine, Wake Forest School of Medicine, Medical Center Boulevard, Winston-Salem, NC 27157, USA; jbolande@wakehealth.edu; 3 Engineering Research Center in Biomaterials, Sichuan University, Chengdu 610064, China; hluo@scu.edu.cn; 4 Center for Nanofibers and Nanotechnology, National University of Singapore, Singapore 117581, Singapore

**Keywords:** retinal pigment, Bruch’s membrane, nanofibrous scaffolds, drug delivery, stem cell therapy

## Abstract

Progenitor cells derived from the retinal pigment epithelium (RPECs) have shown promise as therapeutic approaches to degenerative retinal disorders including diabetic retinopathy, age-related macular degeneration and Stargardt disease. However, the degeneration of Bruch’s membrane (BM), the natural substrate for the RPE, has been identified as one of the major limitations for utilizing RPECs. This degeneration leads to decreased support, survival and integration of the transplanted RPECs. It has been proposed that the generation of organized structures of nanofibers, in an attempt to mimic the natural retinal extracellular matrix (ECM) and its unique characteristics, could be utilized to overcome these limitations. Furthermore, nanoparticles could be incorporated to provide a platform for improved drug delivery and sustained release of molecules over several months to years. In addition, the incorporation of tissue-specific genes and stem cells into the nanostructures increased the stability and enhanced transfection efficiency of gene/drug to the posterior segment of the eye. This review discusses available drug delivery systems and combination therapies together with challenges associated with each approach. As the last step, we discuss the application of nanofibrous scaffolds for the implantation of RPE progenitor cells with the aim to enhance cell adhesion and support a functionally polarized RPE monolayer.

## 1. Introduction

The most prevalent retinal degenerative disorders include diabetic retinopathy (DR) and age-related macular degeneration (AMD) all around the world [1]. Due to a lack of reliable treatment alternatives, these conditions often lead to visual impairment or even blindness. AMD currently represents the main cause of blindness that affects 200 million people and may increase to 280 million by 2040 [1]. AMD is divided into two types, dry (atrophic) and wet (neovascular or exudative) [2]. The dry form of AMD is characterized by an accumulation of membranous debris containing fats and proteins between the retinal pigment epithelium (RPE) and Bruch’s membrane (BM), known as druses, which induces local inflammation [3]. In the case of the wet AMD, degeneration of the RPE and a reduced number of photoreceptor cells is observed due to permeable choroidal blood vessels, which cause the increase of macrophages in the RPE [4]. Although the mechanism remains largely unknown, both the dry and wet form of AMD lead to the dysfunction and degeneration of RPE, which eventually causes the loss of central vision [5]. Most AMD starts as the dry type, and in 10–20% of individuals progresses into the wet type [5]. Similar to AMD, there are retinal degenerative diseases including retinitis pigmentosa (RP), which is a monogenic disease that causes vision loss due to dysfunction of RPE cells [5]. Cell replacement therapy has beneficial properties for irreversible retinal cell death in several diseases, including Stargardt’s disease, AMD, RP and glaucoma [5]. The deposition of lipofuscin-like substances in the RPE, as well as photoreceptor cell death in the macula, are considered a major causes of failure from hereditary Stargardt’s disease [6,7]. Glaucoma is the result of oxidative stress, glutamate toxicity, and alteration in glial cell function [7]. Findings from classical histological studies have demonstrated the pathological changes in high myopia associated with lack of RPE and Bruch’s membrane defects [8]. The estimated global prevalence of myopia will rise to 50% by 2050, especially in several parts of East Asia [9]. Although therapeutic drugs for controlling myopia progression have been utilized, such as atropine and pirenzepine, molecular mechanisms for their therapeutic actions persist as the subject of debate [10]. Since the retina is a fragile light-sensing tissue, promoting alternative strategies to healing damaged tissues of the eye and returning its functions plays an essential role for the degenerative disease [11].

Unfortunately, retinal degeneration is an irreversible procedure, and there is no reliable treatment accessible. The current cure mainly targets neovascularization in order to reduce the development of vision loss and disease symptoms [7]. Anti-VEGF agents such as pegaptanib, ranibizumab, bevacizumab and aflibercept are often utilized to target neovascularization [12]. However, pharmacological therapy for posterior eye segment diseases may be influenced by the efficacy of the drug to reach the target site of action due to anatomical barriers and the poor clearance properties of the eye [13]. Consequently, the combination of novel drug delivery systems with cell-based therapeutics offers a promising option for successful ocular disease therapy. Specifically, the application of nanofibrous scaffolds that mimic natural tissue provides a disease-tunable platform for the treatment of degenerative retinal disease [14,15]. The main purpose of this review is to summarize and highlight current and effective strategies for the treatment of common ocular diseases including nanotechnology-based gene and drug delivery, stem cell-based therapies and the application of engineered nanofibers.

## 2. Gene Delivery Carriers to the Retina

### 2.1. Novel Gene Delivery Systems

**Figure 1:**
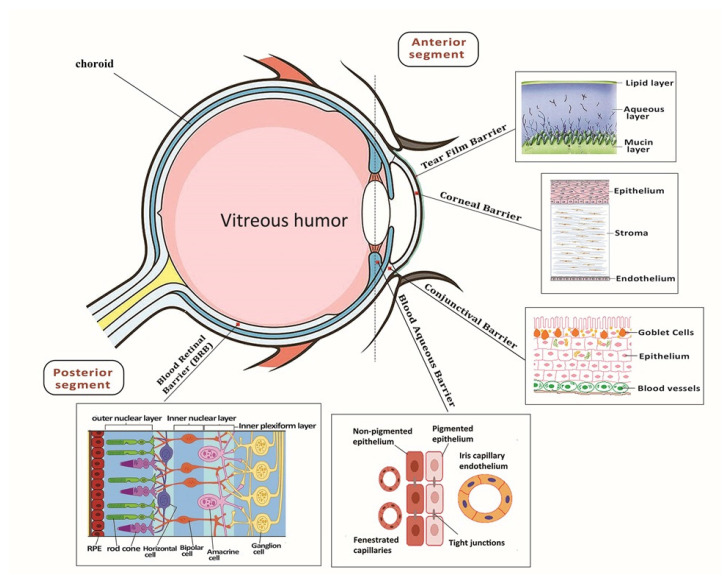
Depiction of eye anatomy and its associated barriers. It can be classified into two segments, anterior segment and posterior segment, which occupy one-third and two-thirds, respectively. The tear film, cornea and conjunctiva have limited diffuse topical delivery. The posterior segment is composed of the sclera, choroid and retina.

Anatomically, the unique segment of the eye includes three layers [16,17]. Meanwhile, there are three chambers with different structures including the anterior chamber that is located between the iris and the cornea, the posterior chamber, which lies between the iris and the lens, and the vitreous chamber covering the lens back to the retina [18]. The major diseases that lead to blindness affect the posterior eye segment where dysfunctional retina cells are a major cause of vision loss [19]. The retina includes a large number of cell types with high intercellular connections and neural projections that play a particular role in the visualizing process (Figure 1) [13].

The RPE cells execute crucial retinal functions including transport of nutrients, absorption of scattered light, regulation of the retinal cycle, phagocytosis of the photoreceptor outer segment (POS) and secretion of growth factors [20]. Interconnections among photoreceptors and RPE cells provide metabolite transport, nutrient, ion, fluid, polarized secretion of cytokines and promote homeostasis between the photoreceptors and the choroid [20]. It is noteworthy that damage to mitochondria can be observed extensively in the RPE of AMD patients, causing lipofuscin due to oxidative stress within the RPE cell populations [21].

Transferring genes and gene replacement therapy for therapeutic purposes are growing approaches for treating some genetic retinal diseases and hereditary eye diseases such as congenital glaucoma, congenital cataract, retinitis pigmentosa (RP), retinoblastoma (RB), congenital corneal dystrophy, Leber congenital amaurosis (LCA) and Usher syndrome [24]. However, prevalent retinal diseases involve a combination of genetic and environmental factors including age-related macular degeneration and diabetic retinopathy. Genetic mutations in specific genes increase the risk of developing the disease [25]. Gene delivery systems have been widely applied to develop gene therapy classified to viral and nonviral vectors [26]. Local delivery to the retina can increase half-life therapeutic agents and reduce immune reactions, while direct retinal gene/drug delivery is a remarkably invasive procedure [27]. Different studies have been demonstrated in preclinical trials using Adeno-associated virus (AAV)-based therapies for Stargardt disease and juvenile retinoschisis [27]. Moreover, a recent novel clinical trial with the title “Retinal Gene Therapy for Choroideremia Using an Adeno-associated Viral Vector (AAV2) Encoding Rab-escort Protein-1 (REP1)” has been submitted to ClinicalTrials.gov (ClinicalTrials.gov Identifier: NCT02077361).

The main components of the BM, which are located between the retina and choroid, are fibronectin, elastin, collagen I and IV, laminin and lipoprotein, which all contribute to the support and the physiological function of RPE cells [22]. As the body ages, the BM changes in ways such as increased thickness due to a higher level of collagen cross-linking, which leads to the accumulation of waste and extracellular materials [23]. Several labs have developed a plasma-modified polydimethylsiloxane sheet with a laminin coating in order to create a biomimetic BM [14]. This model could allow for easier testing of therapeutic approaches to AMD.

Size, charge, and surface modification of nanoparticles could affect uptake by RPE [28]. Gold nanoparticles have higher transfection efficiency as well as an easy synthesis process, and their biocompatibility [25]. For example, Trigueros et al. showed that the transfection efficiency of a green fluorescent protein (GFP) reporter gene wrapped in gold nanoparticles to human retinal pigment epithelium cells was higher than DNA-liposome complexes in vitro and in vivo [29]. Besides, polymer-based NPs can facilitate cell entry through modification of their surface with ligand, antibody and aptamers. Polymers such as poly-lactic-co-glycolic acid (PLGA), albumin and gelatin have been FDA-approved for gene/drug delivery in humans [30]. Singh et al. used PLGA nanoparticles to deliver an anti-VEGF plasmid via an intravitreal pathway as a treatment strategy for retinal neovascular disease [28]. Indeed, hybrid polymer NPs improve gene delivery through endosome escape and higher encapsulation efficiency. A PLGA-chitosan hybrid NP was evaluated by Jin and colleagues for anti-VEGF plasmid delivery into rat retina. Their result confirmed that hybrid chitosan NPs have higher transfection efficiency compared to PLGA alone [31]. Recently, CRISPR/Cas9 has been used as a promoting strategy for monogenic inheritance. For instance, Patrizi et al. showed the curing potential of AAV-Cas9 gene editing to inactivate the human mutation gene retinitis pigmentosa (RP) (Pro347Ser) in a transgenic mouse model [32]. Notably, AAV-Cas9 could remove the mutation of c.2991 + 1655A > G in intron 26 of the CEP290 gene, the most frequent mutation in LCA, through a single-dose of an AAV vector containing three components: an S. aureus Cas9 and two gRNAs–gRNA-323 and gRNA-64. AGN-151587 to treat LCA (ClinicalTrials.gov Identifier: NCT03872479) [33,34]. Furthermore, the exosomes have more advantages compare to SCs for repairing tissue engineering, including high stability for the long term without alteration of biological activity. They can target organs immediately and initiate tissue repair, and preserve a variety of bioactive components from degradation by enzymes [35]. In recent years, studies on therapeutic effects of exosomes as carriers for gene delivery have been evaluated to maintain retinal homeostasis via immune-modulation for retinal degenerative diseases [36]. In the future, the use of exosomes as the carrier for CRISPR/Cas9 technology will remarkably promote the research of genetic disorders and retinal therapy [37].

### 2.2. Novel Drug Delivery Systems

**Figure 2:**
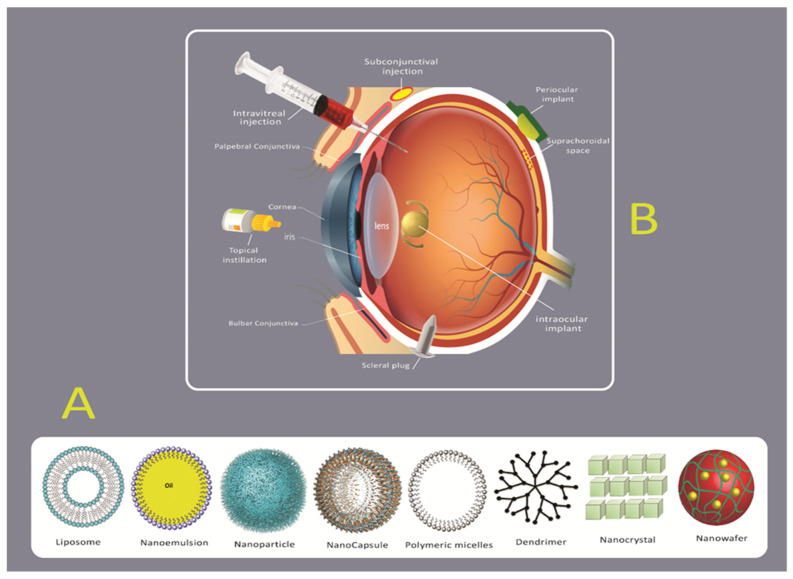
(**A**) Illustration of different types of formulation such as liposomes, nanocarriers, emulsions, nanocapsule, polymeric nanoparticles, dendrimers, nanocrystal and nanowafers for drug delivery into the eye. (**B**) Schematic representation of different strategies for drug delivery to the posterior segment of the eye through topical administration, subconjunctival injection, subretinal injection and intravitreal injection.

Traditional drug delivery strategies to the eye, such as eye drops, are very inefficient at targeting diseased tissue within the posterior eye because of its anatomical location and physiological barriers. Therefore, diseased tissues in confined posterior locations of the eye face challenges due to their anatomical location and physiological barriers (Figure 2) [38]. There are currently four routes for drug delivery to the posterior tissues of the eye: intraocular, topical, systemic and periocular (including retrobulbar subconjunctival and subtenon) [39]. Local administration does not provide the delivery of significant levels of therapeutic agents due to the low leakiness of the blood-aqueous barrier and the corneal epithelium (Figure 1) [39]. Alternatively, systemic administration can deliver a high level of therapeutic agents, but the blood-retinal barrier (BRB) limits those that can be delivered due to its function of regulating substance permeation from the blood to the retina. For this reason, the more common routes of administration are periocular and intravitreal injections [11].

The periocular route enables molecules to be deposited directly against the external surface of the sclera, thereby decreasing the risk of endophthalmitis and retinal damage connected to the intravitreal route of administration [25]. With intravitreal injection, the therapeutic moiety is injected directly into the posterior segment of the eye [25]. However, a distinct advantage of intravitreal injection is that it allows molecules to be directly inserted into the vitreous which avoids the complications associated with passing through the sclera [40]. To minimize the side effects of intravitreal injections, which include retinal endophthalmitis, retinal detachment, increased intraocular pressure and retinal hemorrhages, novel nanoparticle drug delivery systems can be utilized in order to bypass the cornea-conjunctiva barrier [41]. Current research suggests that novel site-specific drug delivery systems can be of great promise for treating diseases of the posterior eye segment (Figure 2) [42].

In an approach to enhance efficacy and reduce the side effects of drug entrance to the intraocular tissues, some researchers are utilizing nanoparticles as drug delivery systems [43,44]. Nanoparticles have many advantages, such as a controlled and prolonged release of drugs to the target site, the ability to pass through blood-optical tissue barriers, decreased time of administration, prevention of drug inactivation by binding tear proteins or lacrimal enzymes and prolonged-time of drug presence in the precorneal area [45]. Nanoparticles also offer the ability for ligand attachment, which can enhance corneal permeation [45,46,47,48]. Moreover, the use of modified nanoparticles for tissue-specific penetration and prevention of degradation of therapeutic agents by more effective routes of delivery, could help develop nonviral vectors with highly efficient transfection activity to the target tissues/cells [35,49]. The positive charge and hydrophobicity characteristic of most drugs is associated with decreased intravitreal mobility. This indicates that nanoparticle surfaces can be decorated with polyethylene glycol (PEGylation) and hyaluronic acid (HA) to render efficient retinal cell uptake [50]. Coating nanocarriers with HA increased the efficiency of retinal gene therapy via intravitreal administration [51,52]. Accordingly, the promoting strategies of drug delivery systems, such as drug loaded implants, microspheres, nanoparticles and liposomes, gels, and transporter-targeted prodrugs, has led to new approaches for the therapeutic of posterior segment diseases (Figure 2) [53,54]. Common biodegradable drug-loaded implants include polycaprolactone (PCL), poly (lactic-co-glycolic acid) (PLGA), poly(orthoester) (POE) and polyanhydride [55,56]. Vitrasert^®^ and Retisert^®^ are two surgical implants that are in clinical use for the treatment of cytomegalovirus retinitis [11,57]. Furthermore, brinzolamide was also investigated as the target compound in nano eye-drops for glaucoma due to improved penetration into the eye via cornea [58]. Another promising drug delivery system for the posterior eye segment is cyclodextrin-based nanoparticle formulations in eye drops [59]. Cyclodextrin nanoparticle eye drops of dexamethasone are under comprehensive research in phase 2/3 clinical trial for curing diabetic macular edema (ClinicalTrials.gov Identifier No. NCT01523314) [59]. Synthetic phospholipids are also commonly utilized as a drug delivery system, which includes phosphatidylcholine derivatives, phosphatidylethanolamines, phosphatidylserines, phosphatidic acids and PEGylated phospholipids [60,61]. Additionally, liposome delivery systems are applicable for encapsulation of drugs and small unstable siRNA molecules without degradation and loss of bioactivity. Among lipid-based ocular drug delivery systems, solid lipid nanoparticles (SLN) and nanostructured lipid carriers (NLC) are attractive because they improve corneal penetration of the drug, enhance the ocular bioavailability, prolong the time of ocular retention and control the drug release profile of both hydrophilic and lipophilic drugs [62,63]

Moreover, a nanowafer DDS has been developed in which the polymer and the drug cooperate in providing enhanced therapeutic efficacy and reduced immune responses [53]. The nanowafer was fabricated from polyvinylpyrrolidone (PVP), polyvinyl alcohol (PVA), hypromellose (HPMC) and carboxymethyl cellulose (CMC) polymers in a circular disk format which contained drug arrays that resulted in controlled release for a few hours to days [53].

Meanwhile, inorganic nanoparticles such as silicon nanoparticles, gold nanoparticles, titanium oxide nanoparticles, magnetic nanoparticles and zinc oxide nanoparticles play an important role in retinal drug delivery [64]. For example, combination hyaluronan with gold nanoparticles can enhance the mobility of the nanoparticles in addition to targeting them to HA receptors due to higher expression in different cells of the eye. The result showed the antiangiogenic effect of gold nanoparticles (GNPs) could inhibit deposits of advanced-glycation end-products (AGEs)-mediated- retinal pigment epithelial cell death [65].

Combination therapy can exert synergistic effects and bring about more efficacious therapies, since complex tissues can be targeted. For instance, polyplexes containing 1,2-dioleoyl-3-trimethylammonium-propane (DOTAP) and the fusogenic lipid, 1,2-dioleoyl-sn-glycero-3-phosphoethanolamine (DOPE) coated with HA have shown more efficient cellular uptake and, consequently, higher transfection efficiency compared to polyplex only [66,67]. A strategy to overcome current hurdles could be to optimize particle design for specific targeting of the retina in order to improve the treatment modality. For example, several studies have indicated that intravitreally-injected nanoparticles can be prevented from reaching the retina through vitreous humor [66] Evidence has also shown that microelectronic retinal implants cannot effectively stimulate neurons and that nanocarriers alone cannot result in suitable outcomes for retinal diseases. Thus, combination therapy with stem cells could be considered an alternative and better solution for solving this issue [67]. Won et al., used coaxial printing, making, from a polymeric shell PCL and a hydrogel core PLGA for kinetics release, two types of drugs (bevacizumab (BEV) and dexamethasone (DEX) from a single implant. This implant can inhibit inflammatory responses and suppression of neovascularization for the cure of numerous kinds of retinal vascular diseases [68]. A coculture system can provide intact communication between different cells which may lead to accelerated wound healing [14,69].

### 2.3. Stem Cells and Their Clinical Potential for Retinal Diseases

Since the retina and retinal pigmented epithelium (RPE) have the low capability of regeneration, stem cell therapy to replace lost retinal cells and restoring remaining cells is the critical issue [70].

Stem cells for ocular treatment have been divided into two main group: (1) ocular-derived progenitor cells, including retinal progenitor cells (RPCs) and (2) nonocular-derived stem cells (with the ability to self-renew and produce several cells including RPE, photoreceptors, etc.), including pluripotent stem cells (iPSCs), embryonic stem cells (ESCs) mesenchymal, stromal cells (MSCs) (particularly bone marrow mesenchymal stromal cells (BM-MSCs) and adipose-derived stromal cells (ADSCs) [70]. RPC cells, among all cell types that have been used, are among the best options due to safety, simplicity and accessibility [70]. They are able to differentiate into numerous retinal cell types at different times (rod photoreceptors, Müller glial cells, and bipolar neurons) [70]. Two fundamental cell-based therapy applications include: (1) exploiting cells as a support system for protecting photoreceptors and visual function spatially in retinal degenerative diseases (i.e., AMD and RP) and (2) differentiating stem cells into RPE cells for substituting dead or defective endogenous RPE cells. In terms of regenerative medicine, the latter provides a pivotal step by replacing the apoptotic RPE cells with new functional cells that can integrate onto the BM and restore the function of the tissue [71]. Three specific sources of MSCs have been used in cell-based therapy for retinal diseases including bone marrow-derived MSCs (BM-MSCs), umbilical cord-derived MSCs (UC-MSCs) and neural stem/progenitor cell (NSCs/NPCs) [1,72].

Induction of MSCs to NSCs in a monolayer culture needs a growth factor such as basic fibroblast growth factor (bFGF), epidermal growth factor (EGF), N2, and B27 supplements, forskolin, dimethyl sulfoxide (DMSO), ascorbic acid ad nerve growth factor (NGF) or retinoic [73]. Besides, NSCs are secreted by neurotrophic growth factors (NTF) leading to the restoration of dysfunctional neuronal cells and synapses that could be useful for neurodegenerative diseases [73].

One important advantage of BMSCs is that they regulate immune cells. Especially, they can inhibit M1 macrophage polarization and promote M2 polarization in an extracellular matrix-dependent manner [72]. Compared with UC-MSCs and BM-MSCs, NSCs/NPCs cannot be differentiated into mature retinal cells and, instead, they are involved in the preservation of the photoreceptor through the secretion of neurotrophic factors and photoreceptor outer segment phagocytize [74].

Intravitreal administration is the best delivery method of MSCs for retinal degeneration because of reduced retina damage. Furthermore, secreted factors of MSCs may reach the retina even while the inner restricted membrane prevents MSCs migration [75]. In this way, embryonic stem cell ESCs can also be used as vehicles for the delivery of agents to damaged tissue [76,77]. As an example, MSCs have been shown to efficiently and specifically carry drug-loaded polymeric nanoparticles by efflux transporters, such as overexpressed P-glycoprotein, since they are highly drug resistant [78]. Several studies successfully introduced siRNAs and other transgenes into MSCs while maintaining their proliferation ability and their capability to differentiate into different mesodermal lineages (bone, cartilage and fat) [78]. Stem cells can generate various types of cells affected by the disease, and they do not express major histocompatibility complex class II (MHCII). Consequently, they may possess the potential to restore vision by replacing dead photoreceptors and RPE cells in atrophic AMD and RP, and dying retinal ganglion cells (RGCs) in glaucoma and other ocular nerve atrophies [79]. Alternatively, there are limitations to the promise of stem cells, and yet there are no FDA-approved stem cell therapies for retinal disease [80]. Experimental studies have shown that MSCs differentiated into RPE-like cells with similar morphological features that could replace damaged cells when applied to damaged retinas, and this retreatment was well tolerated, with no intraocular inflammation or proliferation [81]. Cell transplantation, as well as new drug delivery approaches, have been studied for the treatment of retinal degenerative disease [82]. However, the method for cell delivery and maintenance are the current major limitations; the method for cell delivery and maintenance is difficult [47]. Improved attachment of embryonic retinal explants via biodegradable elastomeric membranes containing poly(glycerol-co-sebacic acid) (PGS) laminin and PCL have been accomplished [83]. Although stem cells can differentiate into retinal precursors, combination stem cells with the CRISPR/Cas9 system is a novel strategy for the therapy of genetic mutations [84].

### 2.4. Tissue Engineering and Nanofibrous Scaffolds for Retinal Diseases

**Figure 3:**
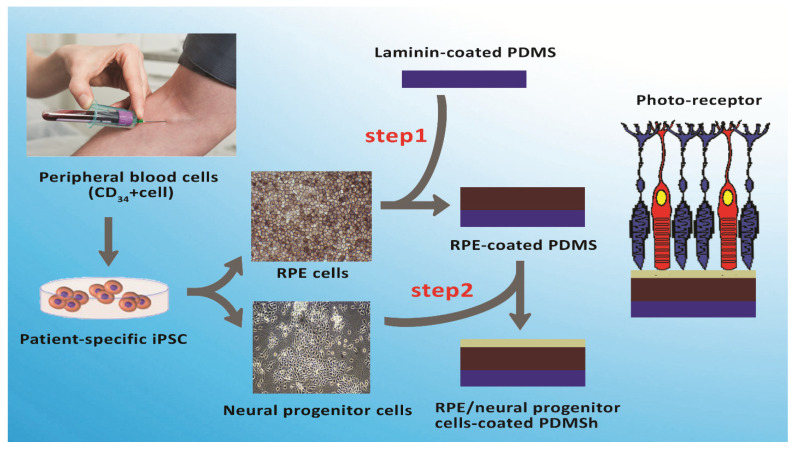
Process of pluripotent stem cells (iPSC) differentiation to photoreceptor and retinal pigment epithelium (RPE) trough bilayer-coating polydimethylsiloxane (PDMS)-PmL biomimetic film. Adapted with permission from [14].

Stem cell therapy for retinal disease faces some challenges, including inefficient RPE cell differentiation that is mostly short-term, and host inflammatory responses which should be avoided by growing RPE cells in a scaffold prior to transplantation [74]. As a multidisciplinary field, tissue engineering aims to restore normal function to dysfunctional tissues by mimicking the structure of natural tissues based on different cells and scaffolds [11,78]. Scaffolds provide the space for cells to proliferate and differentiate. Scaffold stiffness can impact the cells through mechanical cues and also affects the nutrient diffusion necessary for cell survival [85]. Several suitable biomaterials have been utilized to make scaffolds for RPE delivery including natural (collagen, gelatin, etc.), synthetic and hybrid polymers [11]. Synthetic polymers including polylactic acid (PLA), PCL, polyurethanes, poly-L-lactic acid (PLLA), poly (lactic-co-glycolic acid), poly(3-hydroxybutyrate), poly (ethylene glycol) diacrylate (PEGDA) and polydimethylsiloxane (PDMS) are commonly used to produce nanoscaffolds due to their reproducibility and ability to be easily modified (Table 1) (Figure 3) [14,86,87]. Despite the fact that they have shown in vitro success, there are still limitations that must be developed for the implantation of cell-seeded scaffolds. Firstly, following scaffold implantation, interleukin 6 (IL-6) and monocyte chemo-attractant protein-1 (MCP-1) are expressed highly in the retina, which triggers microglia that eventually form a microglial scar on the retinal side of the scaffold [5]. Secondly, RPE cells can lose the functionality of a mature RPE cell due to their ability to dedifferentiate or transdifferentiate into a macrophage or fibroblast-like phenotype via the SMAD3 pathway [88]. Therefore, surface modification methods such as plasma treatment, alkaline hydrolysis or conjugation of cell adhesion protein sequences (RGDs) may accelerate cell adhesion and interaction of cells with materials by topographically and functionally altering the surface [88]. Peng CH et al. demonstrated that oxygen plasma-treated PDMS coated with laminin mimics BM to augment the adherence level of a functional human pluripotent stem cell-differentiated RPE cells monolayer [14]. Interestingly, nanofibers, nanoparticles and nanotubes were extensively reported for manipulating stem cell fate, from which nanofibers are used more than others [89]. It has been reported that chitosan graft PCL and plain PCL in a 20:80 ratio have a higher ability to differentiate murine RPCs to retinal neurons than those cells cultured on PCL scaffolds [90]. Electrospun poly (L-lactic acid-co-ε-caprolactone) (PLCL) scaffolds were capable of enhancing RPE cell proliferation. Furthermore, silk fibroins (SF) are also widely used in tissue engineering because they are biocompatible and permeable to oxygen/water, possess various mechanically stable molecular structures with controllable morphology and they exhibit a low inflammatory response [91,92]. Zhang et al. demonstrated that copolymer SF:PLCL nanofibrous scaffolds can promote RPC development and differentiation via photoreceptors [93]. Hybrid scaffolds have been indicated to support the proliferation and differentiation toward neuronal lineage, thus making them potential materials for retinal tissue repair [94]. Another study showed that PCL and a gelatin scaffold can be utilized as substrates to replace RPE ECM, thereby facilitating the regeneration of the RPE layer affected by retinal diseases [95]. Krishna L. et al. indicated that the diameter of PCL nanofiber scaffolds can affect differentiation of epithelial cells of ocular origin [96]. Their results revealed that epithelial cells (HCE-T) express differentiation and lower apoptotic markers on PCL nanofibers of 500 nm, whereas with nanofibers of 1300 nm the cells revealed increasing expression of the corneal stem/progenitor as well as pluripotent stem cell markers [96].

Core-shell fibrous scaffolds have been utilized for the controlled release of a wide range of materials such as growth factors and drugs. Core-shell aligned fibrous scaffolds (PEG /PCL) have been used for sustained release of retinal pigmented epithelium-derived factor (PEDF) to stimulate differentiation of conjunctiva mesenchymal stem cells (CJMSCs) seed on scaffolds to photoreceptors [97].

Furthermore, using a hydrogel for cell delivery into the subretinal space was investigated, especially fibrin gel. In other words, fibrin is a major compartment of the extracellular matrix (ECM), and fibrin gel moderates cell adhesion, motion and mediates cell proliferation and differentiation [98]. Using a hydrogel of gelatin (Ge)/gellan gum (GG)/glycol chitosan (CS) for encapsulating ARPE-19 cells indicated the Ge/GG/Cs could enhance the microenvironment for cell−cell and cell−matrix interaction, and the expression of RPE-specific proteins and genes increased in the Ge/GG/CS matrix. Although the amount of CS changed the morphology of the Ge/GG matrix, it improved the mechanical feature of the hydrogel and the stability of the matrix [99].

## 3. Conclusions

Although nanocarriers have shown effective drug delivery potential to the anterior eye segment, they are associated with serious side effects for posterior eye segment delivery and are limited in their use due to physiological barriers. The combination of delivery approaches, such as codelivery of cells and drugs, would be expected to improve drug release for longer periods compared to traditional methods. However, cells need supportive membranes for successful transplantation. This review article has provided an overview of the production of several desirable scaffolds that have indicated major achievements for retinal diseases. Nanofibrous scaffolds provide appropriate platforms for cell migration through the scaffold and lead to the formation of a cell monolayer on top, while surface modifications to the nanofibers improve cell adhesion. Moreover, nanofibrous scaffolds can promote cell proliferation and differentiation.

## 4. Future Perspective

Future studies must corroborate the reported findings/hypotheses and improve the understanding of the novel ultrathin three-dimensional nanofibrous membranes, which can mimic the fibrillar structure of the native inner layer of collagens in human BM. Meanwhile, combination therapies with multimodal delivery systems provide more advantages in the treatment of retinal diseases [27]. For example, nanosystems such as nanosheets can promote the stability of the monolayer of RPE cells. Thus, more investigation that considers the codelivery of cells and drugs to prevent inflammatory and immune responses will be beneficial in retinal therapy.

## Figures and Tables

**Table 1:** Nanomaterial scaffolds applied for ocular tissue engineering.

Scaffolds	Components	Stem Cells
Nanofibrous scaffolds [100]	Polycaprolactone (PCL)	hRPE
Hybrid nanofibrous scaffolds [87]	Collagen type I and poly(lactic-*co*-glycolic acid) (PLGA)	hRPE
Hybrid nanofibrous scaffolds [90]	Chitosan-graft-poly(ε-caprolactone)/polycaprolactone (CS-PCL/PCL)	RPCs
Hybrid nanofibrous scaffolds [93]	Silk fibroin, PCL, and Gelatin	iPSCs
Hybrid nanofibrous scaffolds [92]	Laminin and Poly(epsilon-caprolactone) (PCL)	RSC
Hybrid nanofibrous scaffolds [93]	Silk fibroin (SF) and Poly(L-lactic acid-co-ε-caprolactone) (PLCL)	RPCs
Nanofibrous scaffold [96]	Polycaprolactone (PCL)	HCE-T
Core-shell nanofibrous scaffolds [97]	(PEG /PCL)	CJMSCs
Hydrogel scaffold [99]	the hydrogel of gelatin (Ge)/gellan gum (GG)/glycol chitosan (CS)	ARPE-19

## Data Availability

Data is contained within the article.
